# Generation of an induced pluripotent stem cell line (TRNDi005-A) from a Mucopolysaccharidosis Type IVA (MPS IVA) patient carrying compound heterozygous p.R61W and p.WT405del mutations in the *GALNS* gene

**DOI:** 10.1016/j.scr.2019.101408

**Published:** 2019-02-15

**Authors:** Rong Li, Amanda Baskfield, Jeanette Beers, Jizhong Zou, Chengyu Liu, Carlos J. Alméciga-Díaz, Wei Zheng

**Affiliations:** a National Center for Advancing Translational Sciences, National Institutes of Health, Bethesda, MD, USA; b iPSC core, National Heart, Lung and Blood Institute, National Institutes of Health, Bethesda, MD, USA; c Transgenic Core, National Heart, Lung and Blood Institute, National Institutes of Health, Bethesda, MD, USA; d Institute for the Study of Inborn Errors of Metabolism, Faculty of Sciences, Pontificia Universidad Javeriana, Bogotá, Colombia

## Abstract

Mucopolysaccharidosis type IVA (MPS IVA) is a rare genetic disease caused by mutations in the *GALNS* gene and is inherited in an autosomal recessive manner. *GALNS* encodes *N*-acetylgalactosamine-6-sulfatase that breaks down certain complex carbohydrates known as glycosaminoglycans (GAGs). Deficiency in this enzyme causes accumulation of GAGs in lysosomes of body tissues. A human induced pluripotent stem cell (iPSC) line was generated from dermal fibroblasts of a MPS IVA patient that has compound heterozygous mutations (p.R61W and p.WT405del) in the *GALNS* gene. This iPSC line offers a useful resource to study the disease pathophysiology and a cell-based model for drug development.

## Resource utility

This human induced pluripotent stem cell (iPSC) line is a useful tool for studies of disease phenotype and pathophysiology, and use as a cell-based disease model for drug development to treat MPS IVA patients.

## Resource details

Mucopolysaccharidosis type IVA (MPS IVA, also called Morquio syndrome type A) is a rare autosomal recessive disorder caused by mutations in the *GALNS* gene, which encodes *N*-acetylgalactosamine-6-sulfatase (EC 3.1.6.4). It breaks down certain complex carbohydrates known as glycosaminoglycans (GAGs). Deficiency in *N*-acetylgalactosamine-6-sulfatase results in accumulations of GAGs in the lysosomes in body tissues and causes clinical symptoms including heart disease, skeletal abnormalities, vision and hearing loss, difficulty breathing, and early death (Khan et al., 2017; Peracha et al., 2018).

**Fig. 1.**
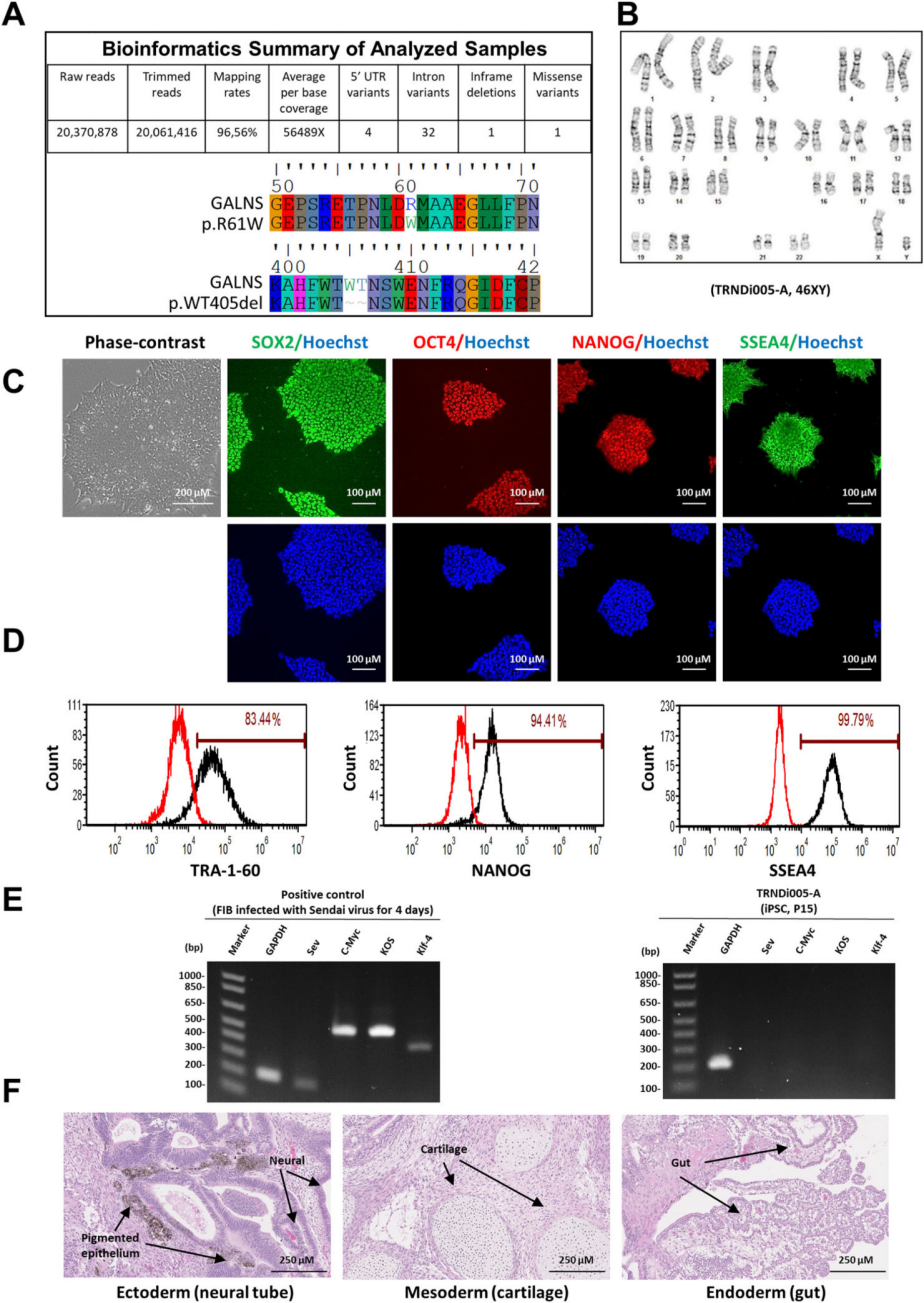
Characterization of TRNDi005-A iPSC line. **A)** Detection of compound heterozygous mutations of p. R61W in exon 2 and p. WT405del in exon 11 of the *GALNS* gene. **B)** Cytogenetic analysis showing a normal karyotype (46, XY). **C)** Left: Phase contrast imaging of TRNDi005-A colonies grown on Matrigel at passage 10. Right: Representative immunofluorescent micrographs of iPSCs positive for stem cell markers: SOX2, OCT4, NANOG, and SSEA4. Nucleus is labelled with Hoechst (in blue). **D)** Flow cytometry analysis of pluripotency protein markers: TRA-1-60, NANOG and SSEA4. **E)** RT-PCR verification of the clearance of Sendai virus from the reprogrammed cells. Sendai virus vector transduced fibroblasts was used as positive control. **F)** Pathological analysis of a teratoma from TRNDi005-A iPSC, showing a normal ectodermal, endodermal and mesodermal differentiation.

In this study, a human iPSC line was established from dermal fibroblasts of a 43-year-old male patient (GM01361, Coriell Institute) carrying compound heterozygous mutations of a p. R61W variant (c.181G > A) in exon 2 and a p.WT405del variant (c. 1213_1218del) in exon 11 of the *GALNS* gene (Table 1, Fig. 1A). A non-integrating CytoTune-Sendai viral vector kit (A16517, Thermo Fisher Scientific) containing OCT3/4, KLF4, SOX2 and C-MYC pluripotency transcription factors was employed to transduce the fibroblasts using the method described previously (Beers et al., 2012; Beers et al., 2015). The resulting iPSC line was named TRNDi005-A that exhibited a classical embryonic stem cell morphology (Fig. 1C), normal karyotype (46, XY), as confirmed by the G-banding karyotype at passage 11 (Fig. 1B), and expressed the major pluripotent protein markers of NANOG, SOX2, OCT4, SSEA4 and TRA-1–60 (Fig. 1C, D) evidenced by both immunofluorescence staining and flow cytometry analysis. Sendai virus vector (SeV) clearance was detected with reverse transcription polymerase chain reaction (RT-PCR) using SeV-specific primers that confirmed the vector elimination in the iPSCs by passage 15 (Fig. 1E). This iPSC line was not contaminated with mycoplasma (Supplementary Fig. S1) and were authenticated using a short tandem repeat (STR) DNA analysis, which demonstrated matching genotypes at all 16 loci examined (information available with the authors). Furthermore, the pluripotency of this iPS cell line was confirmed by a teratoma formation experiment that exhibited its ability to differentiate into cells of all three germ layers (Ectoderm, neural tube and pigmented epithelium; Mesoderm, cartilage; Endoderm, gut) *in vivo* (Fig. 1F).

## Materials and methods

### Cell culture

Patient skin fibroblasts were obtained from Coriell Cell Repositories (GM01361) and cultured in DMEM supplemented with 10% fetal bovine serum, 100 units/ml penicillin and 100 μg/ml streptomycin in a humidified incubator with 5% CO_2_ at 37 °C. The iPS cells were cultured in StemFlex medium (Thermo Fisher Scientific) on Matrigel (Corning, 354,277)-coated plates at 37 °C in humidified air with 5% CO_2_ and 5% O_2_. The cells were dissociated with 0.5 mM Ethylenediaminetetraacetic acid (EDTA) and passaged when they reached 80% confluency.

### Reprogramming of human skin fibroblasts

Patient fibroblasts were reprogrammed into iPS cells using the non-integrating Sendai virus technology following the method described previously (Beers et al., 2012; Beers et al., 2015).

### Genome analysis

The genome analysis of variants in *GALNS* was conducted through ACGT, Inc. (Wheeling, IL, USA). Briefly, genomic DNA was extracted from MPS IVA patient fibroblasts using MasterPure Complete DNA and RNA Purification kit (Epicentre, Madison, WI, USA) as per manufacture instruction, followed by PCR amplification of all the exons using PrimeSTAR GXL DNA Polymerase (Takara, Mountain View, CA, USA). Equal quantities of each PCR amplicon were polled and fragmented to an average of size of 400 bp for library preparation. Sequencing of human *GALNS* exons by Next-Generation Sequencing (NGS) and bioinformatics data analysis was performed to identity the variants in the *GALNS* gene of MPS IVA patient sample. Gene variants were analyzed by using the Variant Annotation Integrator (VAI) tool from the University of California Santa Cruz Genome Browser (Casper et al., 2018). The specific primers for amplification of *GALNS* exons are listed in Table 2.

### Immunocytochemistry

For immunofluorescence staining, patient iPSCs were fixed in 4% paraformaldehyde for 15 mins, rinsed with Dulbecco’s Phosphate Buffered Saline (DPBS), and permeabilized with 0.3% Triton X-100 in DPBS for 15 mins. The cells were then incubated with the Image-iT™ FX signal enhancer (Thermo Fisher Scientific) for 40 mins at room temperature in a humidified environment and then followed by incubation individually with primary antibodies including SOX2, OCT4, NANOG and SSEA4, diluted in the Image-iT™ FX signal enhancer blocking buffer, overnight at 4 °C. After washing with DPBS, a corresponding secondary antibody conjugated with Alexa Fluor 488 or Alex Fluor 594 was added to the cells and incubated for 1 h at room temperature (Antibodies used are listed in Table 2). Cells were then washed and stained with Hoechst 33342 nucleic acid stain for 15 mins and imaged using an INCell Analyzer 2200 imaging system (GE Healthcare) with 20× objective lens and Texas Red, FITC and DAPI filter sets.

### Flow cytometry analysis

The iPSCs were harvested using TrypLE Express enzyme (Thermo Fisher Scientific). Cells were fixed with 4% paraformaldehyde for 10 mins at room temperature and then washed with DPBS. Before fluorescence-activated cell sorting analysis, cells were permeabilized with 0.2% Tween-20 in DPBS for 10 mins at room temperature and stained with fluorophore-conjugated antibodies for 1 h at 4 °C on a shaker. Relative fluorophore-conjugated animal nonimmune Immunoglobulin were used as the negative control (Antibodies and nonimmune immunoglobulin used are listed in Table 2). Cells were then analyzed on a BD Accuri™ C6 Flow Cytometry system (BD Biosciences).

### G-banding karyotype

The G-banding karyotype analysis was conducted at WiCell Research Institute (Madison, WI, USA). A total of 20 randomly selected metaphases were analyzed by G-banding for each cell line.

### Short tandem repeat (STR) analysis

Patient fibroblasts and derived iPSC lines were sent to the WiCell Institute for STR analysis. Briefly, the Promega PowerPlex® 16 HS System (Promega, Madison, WI) was used in multiplex polymerase chain reaction (PCR) to amplify fifteen STR loci (D5S818, D13S317, D7S820, D16S539, vWA, TH01, TPOX, CSF1PO, D18S51, D21S11, D3S1358, D8S1179, FGA, Penta D, Penta E) plus a gender determining marker, Amelogenin (AMEL). The PCR product was capillary electrophoresed on an ABI 3500xL Genetic Analyzer (Applied Biosystems) using the Internal Lane Standard 600 (ILS 600) (Promega, Madison, WI). Data were analyzed using GeneMapper® v 4.1 software (Applied Biosystems).

### Mycoplasma detection

Mycoplasma testing was performed and analyzed using the Lonza MycoAlert kit following the instructions from the company. Ratio B/A > 1.2 indicates mycoplasma positive. Ratio B/A 0.9–1.2 indicates ambiguous results and Ratio B/A < 0.9 indicates mycoplasma negative.

### Testing for Sendai reprogramming vector clearance

Total RNA was extracted from TRNDi005-A iPSCs at passage 15 using RNeasy Plus Mini Kit (Qiagen). Human fibroblasts (GM05659, Coriell Institute) after transduction with Sendai virus for 4 days was used as the positive control. A total of 1 μg RNA/reaction was reverse transcribed with SuperScript™ III First-Strand Synthesis SuperMix kit and PCR was performed using Platinum II Hot-Start PCR Master Mix (Thermo Fischer Scientific). The amplifications were carried out using the following program: 94 °C, 2 mins; 30 cycles of [94 °C, 15 s, 60 °C, 15 s and 68 °C, 15 s] on Mastercycler pro S (Eppendorf) with the primers listed in Table 2. The products were then loaded to the *E*-Gel® 1.2% with SYBR Safe™ gel, run at 120 V electric field, and then imaged by G: Box Chemi-XX6 gel doc system (Syngene, Frederick, MD).

### Teratoma formation assay

Patient iPSCs cultured in 6- well plates were dissociated with DPBS containing 0.5 mM EDTA and approximately 1 × 10^7^ dissociated cells were resuspended in 400 μl culture medium supplied with 25 mM HEPES (pH 7.4) and stored on ice. Then, 50% volume (200 μl) of cold Matrigel (Corning, 354277) was added and mixed with the cells. The mixture was injected subcutaneously into NSG mice (JAX No. 005557) at 150 μl per injection site. Visible tumors were removed 6–8 weeks post injection, and were immediately fixed in 10% Neutral Buffered Formalin. The fixed tumors were embedded in paraffin and stained with hematoxylin and eosin.

## Supplementary Material

supplemental material

## Figures and Tables

**Table 1.** Characterization and validation.

Classification	Test	Result	Data

Morphology	Photography	Normal	Fig. 1 Panel C
Phenotype	Immunocytochemistry	SOX2, OCT4, NANOG, SSEA-4	Fig. 1 Panel C
	Flow cytometry	TRA-1-60 (83.44%); NANOG (94.41%) SSEA-4 (99.79%)	Fig. 1 Panel D
Genotype	Karyotype (G-banding) and resolution	46XY Resolution: 425–475	Fig. 1 Panel B
			
Identity	Microsatellite PCR (mPCR) OR STR analysis	Not performed	N/A
		16 sites tested, all sites matched	Available from the authors
Mutation analysis (IF APPLICABLE)	Sequencing	Compound heterozygous mutation of *GALNS* p.R61W, p.WT405del	Fig. 1 Panel A
	Southern Blot OR WGS	N/A	N/A
Microbiology and virology	Mycoplasma	Mycoplasma testing by luminescence. Negative	Supplementary Fig. S1
Differentiation potential	Teratoma formation	Teratoma with three germlayers formation. Ectoderm (neural tube, pigmented epithelium); Mesoderm (cartilage); Endoderm (gut-like epithelium)	Fig. 1 Panel F
Donor screening (OPTIONAL)	HIV 1 + 2 Hepatitis B, Hepatitis C	N/A	N/A
Genotype additional info (OPTIONAL)	Blood group genotyping	N/A	N/A
	HLA tissue typing	N/A	N/A

**Table 2.** Reagents details.

Antibodies used for immunocytochemistry/flow-cytometry			

	Antibody	Dilution	Company Cat # and RRID

Pluripotency Markers	Mouse anti-SOX2	1:50	R & D systems, Cat# MAB2018, RRID: AB_358009
Pluripotency Markers	Rabbit anti-NANOG	1:400	Cell signaling, Cat# 4903, RRID: AB_10559205
Pluripotency Markers	Rabbit anti-OCT4	1:400	Thermo Fisher, Cat# A13998, RRID: AB_2534182
Pluripotency Markers	Mouse anti-SSEA4	1:1000	Cell signaling, Cat# 4755, RRID: AB_1264259
Secondary Antibodies	Donkey anti-Mouse IgG (Alexa Fluor 488)	1:400	Thermo Fisher, Cat# A21202, RRID: AB_141607
Secondary Antibodies	Donkey anti-Rabbit IgG (Alexa Fluor 594)	1:400	Thermo Fisher, Cat# A21207, RRID: AB_141637
Flow Cytometry Antibodies	Anti-Tra-1–60-DyLight 488	1:50	Thermo Fisher, Cat# MA1–023-D488X, RRID: AB_2536700
Flow Cytometry Antibodies	Anti-Nanog-Alexa Fluor 488	1:50	Millipore, Cat# FCABS352A4, RRID: AB_10807973
Flow Cytometry Antibodies	anti-SSEA-4-Alexa Fluor 488	1:50	Thermo Fisher, Cat# 53–8843-41, RRID: AB_10597752
Flow Cytometry Antibodies	Mouse-IgM-DyLight 488	1:50	Thermo Fisher, Cat# MA1–194-D488, RRID: AB_2536969
Flow Cytometry Antibodies	Rabbit IgG-Alexa Fluor 488	1:50	Cell Signaling, Cat# 4340S, RRID: AB_10694568
Flow Cytometry Antibodies	Mouse IgG3-FITC	1:50	Thermo Fisher, Cat# 11–4742-42, RRID: AB_2043894

Primers			

	Target	Forward/Reverse primer (5′−3′)	

Sev specific primers (RT-PCR)	Sev/181 bp	GGA TCA CTA GGT GAT ATC GAG C/ACC AGA CAA GAG TTT AAG AGA TAT GTA TC	
Sev specific primers (RT-PCR)	KOS/528 bp	ATG CAC CGC TAC GAC GTG AGC GC/ACC TTG ACA ATC CTG ATG TGG	
Sev specific primers (RT-PCR)	Klf4/410 bp	TTC CTG CAT GCC AGA GGA GCC C/AAT GTA TCG AAG GTG CTC AA	
Sev specific primers (RT-PCR)	C-Myc/523 bp	TAA CTG ACT AGC AGG CTT GTC G/TCC ACA TAC AGT CCT GGA TGA TGA TG	
House-Keeping gene (RT-PCR)	GAPDH/197 bp	GGA GCG AGA TCC CTC CAA AAT/GGC TGT TGT CAT ACT TCT CAT GG	
Targeted mutation analysis (PCR)	GALNS/523 bp	TCC GCG GCT CCC GTG GTT GCC AT/CCA CCT TTC CCA AGA GTA GAG	
Targeted mutation analysis (PCR)	GALNS/3201 bp	CCT TAG CGG AAA TGG ATT CTT G/GGT GGC TTC TTT GTG GTT TAG	
Targeted mutation analysis (PCR)	GALNS/6854 bp	GGT GCT CGT CTT ACC AAG AAT/CTC CAT TTA ACT CAG GAC TGC T	
Targeted mutation analysis (PCR)	GALNS/320 bp	GTG AGC ATG TAT GCA TAT CTG TAG/AGC TCT GGG CTT CAC TAC TT	
Targeted mutation analysis (PCR)	GALNS/2541 bp	AAG TAT CAA CCA AGA CCT CAC G/GGG TAT GAA TAG CAA CAG CAG A	
Targeted mutation analysis (PCR)	GALNS/4377 bp	CTG AGC CTT CAT TTC CTC TCT T/GAA GGC TGA CTG AAC CAA TGT A	

**Table:** Resource table.

Unique stem cell line identifier	TRNDi005-A
Alternative name(s) of stem cell line	HT438A
Institution	National Institutes of Health National Center for Advancing Translational Sciences Bethesda, Maryland, USA
Contact information of distributor	Dr. Wei Zheng, wei.zheng@nih.gov
Type of cell line	iPSC
Origin	Human
Additional origin info	Age: 43-year-old Sex: Male Ethnicity: Caucasian
Cell Source	Skin dermal fibroblasts
Clonality	Clonal
Method of reprogramming	Integration-free Sendai viral vectors
Genetic Modification	NO
Type of Modification	N/A
Associated disease	Mucopolysaccharidosis type IVA (MPS IVA)
Gene/locus	*GALNS*^R61W^; *GALNS*^WT405del^
Method of modification	N/A
Name of transgene or resistance	N/A
Inducible/constitutive system	N/A
Date archived/stock date	06-29-2017
Cell line repository/bank	N/A
Ethical approval	NIGMS Informed Consent Form was obtained from patient at time of sample submission. Confidentiality Certificate: CC-GM-15-004
