# Supplementary material for: Generation of an induced pluripotent stem cell line (TRNDi005-A) from a Mucopolysaccharidosis Type IVA (MPS IVA) patient carrying compound heterozygous p.R61W and p.WT405del mutations in the GALNS gene
Source: Stem Cell Res. Author manuscript; Available in PMC 2021 Apr 14. (PMC8045742; doi:10.1016/j.scr.2019.101408)
Supplement: supplemental material [file NIHMS1603952-supplement-supplemental_material.docx]

**Generation of an induced pluripotent stem cell line (TRNDi005-A) from a Mucopolysaccharidosis Type IVA (MPS IVA) patient carrying compound heterozygous p.R61W and p.WT405del mutations in the *GALNS* gene**

**Authors:**

Rong Li^a^, Amanda Baskfield^a^, Jeanette Beers^b^, Jizhong Zou^b^, Chengyu Liu^c^, Carlos J. Alméciga-Díaz^d*^, Wei Zheng^a*^

**Affiliations:**

^a^ National Center for Advancing Translational Sciences, National Institutes of Health, Bethesda, MD, USA.

^b^ iPSC core, National Heart, Lung and Blood Institute, National Institutes of Health, Bethesda, MD, USA.

^c^ Transgenic Core, National Heart, Lung and Blood Institute, National Institutes of Health, Bethesda, MD, USA

^d^ Institute for the Study of Inborn Errors of Metabolism, Faculty of Sciences, Pontificia Universidad Javeriana, Bogotá, Colombia.

**Supplementary Fig. S1**

|  | **Negative control** | **Positive control** | **TRNDi005-A** |
| --- | --- | --- | --- |
| **Read A** | 3.88 | 4.41 | 0.07 |
|  | 4.63 | 3.53 | 0.08 |
|  | 5.05 | 4.16 | 0.03 |
|  |  |  |  |
| **Read B** | 0.49 | 8.75 | 0.02 |
|  | 0.5 | 65.63 | -0.06 |
|  | 0.64 | 0.64 | -0.03 |
|  |  |  |  |
| **Ratio B/A** | 0.12 ± 0.01 | 10.29 ± 11.74 | -0.54 ± 0.68 |
